# Intersections of food insecurity, violence, poor mental health and substance use among US women living with and at risk for HIV: Evidence of a syndemic in need of attention

**DOI:** 10.1371/journal.pone.0252338

**Published:** 2021-05-26

**Authors:** Anna M. Leddy, Jennifer M. Zakaras, Jacqueline Shieh, Amy A. Conroy, Ighovwerha Ofotokun, Phyllis C. Tien, Sheri D. Weiser

**Affiliations:** 1 Department of Medicine, University of California, San Francisco (UCSF), San Francisco, CA, United States of America; 2 School of Medicine, Emory University and Grady Healthcare System, Atlanta, GA, United States of America; 3 Department of Veteran Affairs Medical Center, San Francisco, CA, United States of America; British Columbia Centre for Excellence in HIV/AIDS, CANADA

## Abstract

**Background:**

Food insecurity and intimate partner violence (IPV) are associated with suboptimal HIV prevention and treatment outcomes, yet limited research has explored how food insecurity and IPV intersect to influence HIV-related behaviors. To fill this gap, we conducted a qualitative study with women living with or at risk for HIV in the United States.

**Methods:**

We conducted 24 in-depth interviews with women enrolled in the San Francisco and Atlanta sites of the Women’s Interagency HIV study (WIHS). Participants were purposively sampled so half were living with HIV and all reported food insecurity and IPV in the past year. Semi-structured interviews explored experiences with food insecurity and IPV, how these experiences might be related and influence HIV risk and treatment behaviors. Analysis was guided by an inductive-deductive approach.

**Results:**

A predominant theme centered on how food insecurity and IPV co-occur with poor mental health and substance use to influence HIV-related behaviors. Women described how intersecting experiences of food insecurity and IPV negatively affected their mental health, with many indicating using substances to “feel no pain”. Substance use, in turn, was described to perpetuate food insecurity, IPV, and poor mental health in a vicious cycle, ultimately facilitating HIV risk behaviors and preventing HIV treatment adherence.

**Conclusions:**

Food insecurity, IPV, poor mental health and substance use intersect and negatively influence HIV prevention and treatment behaviors. Findings offer preliminary evidence of a syndemic that goes beyond the more widely studied “SAVA” (substance use, AIDS, and violence) syndemic, drawing attention to additional constructs of mental health and food insecurity. Quantitative research must further characterize the extent and size of this syndemic. Policies that address the social and structural drivers of this syndemic, including multi-level and trauma-informed approaches, should be implemented and evaluated to assess their impact on this syndemic and its negative health effects.

## Introduction

Food insecurity and intimate partner violence (IPV) are major health issues in the United States (U.S.) [1, 2]. Food insecurity, experienced by an estimated one in nine households in 2018 [2], refers to having limited or uncertain availability of adequate food [3]. Black, Latinx, low-income, and female-headed households are among those that bear the greatest burden of food insecurity [2]. Women living with HIV (WLHIV) are also significantly more affected, with approximately 40% estimated to be food insecure in the U.S. [4]. Lifetime IPV prevalence– 36% for women overall [5]–is disproportionately higher for women of color and WLHIV [1, 6]. IPV can include physical (e.g., beating, choking), sexual (e.g., rape, sexual coercion) or emotional violence (e.g., stalking, threats of violence) perpetrated by a current or former intimate partner [7].

High rates of food insecurity and IPV, particularly among HIV-affected populations in the U.S., have significant public health implications. Both food insecurity and violence are well-established determinants of HIV risk behaviors such as condomless sex, transactional sex, and substance use, [8–11] as well as HIV-related morbidity and mortality [12–16]. For example, food insecure individuals may increase their risk of HIV exposure by engaging in high-risk sex, such as condomless or transactional sex, to access food [10]. IPV in the form of coerced or forced condomless sex can also directly increase transmission risk [17]. Food insecurity can lead to poor HIV control by undermining antiretroviral therapy (ART) adherence if people living with HIV (PLHIV) avoid medication to prevent adverse effects from taking ART on an empty stomach (e.g., nausea, vomiting) [14]. Additionally, PLHIV may need to make trade-offs between spending limited resources on food instead of ART or transportation to clinic appointments, negatively effecting disease management [14, 18]. Reduced engagement in HIV care and treatment can likewise arise from fear of or experiences with IPV. Women who anticipate a violent reaction from their partner upon learning of their HIV status are less likely to link to and remain in HIV care and treatment [19–23]. WLHIV who experience violence are also less likely to adhere to ART [19, 21, 24–34] and achieve viral suppression [25, 35–37].

While a large body of research has demonstrated that food insecurity and IPV are independently associated with worse HIV outcomes, few studies have explored how intersecting experiences of food insecurity and IPV shape HIV risk behaviors and treatment adherence. Past research has found that food insecurity and IPV are associated [38–42], with some evidence of a bi-directional relationship [43, 44]. For example, in a longitudinal study among women in the U.S., Conroy and colleagues found that past and current experiences of food insecurity predicted current experiences of sexual or physical and emotional violence [43]. Another longitudinal study of mothers in the U.S. found that experiences of IPV predicted an increased risk of household food insecurity two years later. [44] A small but growing body of evidence suggests that food insecurity and IPV may be linked through pathways of poor mental health and relationship stress and conflict [44–47]. Specifically, research suggests that depression mediates the relationship between food insecurity and IPV [44], and that food insecurity may increase IPV through elevated household stress and related conflict [44–47]. However, limited research has explored the unique ways in which food insecurity and IPV intersect in the context of HIV, or the mechanisms through which food insecurity and IPV may shape HIV risk and treatment behaviors. Such research is critical given that WLHIV are disproportionately affected by both food insecurity and IPV, and each have been shown to be independently associated with HIV risk behaviors and suboptimal engagement in HIV treatment. It also aligns with recent calls for syndemic approaches to understanding the intersecting experiences and cumulative effects of food insecurity, mental health and domestic violence [48]. Accordingly, we conducted a qualitative study to explore how food insecurity and violence intersect and shape HIV risk and treatment behaviors among women living with and at risk for HIV in the U.S.

### Theoretical framing

This study draws upon Singer’s syndemic theory, which describes the co-occurrence of two or more disease clusters at the population-level, whose interaction exacerbates the negative health impacts of each disease involved [49]. Syndemics can involve health conditions of all types, including infectious and non-communicable diseases and mental health conditions [50]. Importantly, this framework acknowledges that the clustering of such epidemics arise from harmful social and structural contexts, such as poverty, racism, and stigma, which contribute to health disparities [49, 50]. The most widely studied syndemic is the substance abuse, violence and AIDS (SAVA) syndemic, in which substance abuse, violence and HIV/AIDS co-occur and mutually reinforce each other in a manner that magnifies worse health outcomes [35, 49]. Food insecurity remains an understudied element of syndemic theory [51], despite the critical role it plays in the lives of marginalized and low-income populations, including WLHIV. It is important to note that although food insecurity and socio-economic status are related, food insecurity is a distinct construct that warrants its own consideration and examination through a syndemic lens. Indeed, not all low-income individuals in the U.S. experience food insecurity [52]. Furthermore, studies have found that individuals living in wealthier households can experience food insecurity in the context of unequal household food allocation, drought, or poor access to healthful foods in their neighborhood [53–55]. Food insecurity has also been found to be associated with poor health outcomes above and beyond the effects of socioeconomic status [56]. Given the evidence, outlined above, that food insecurity and IPV are associated with each other and independently associated with poor HIV outcomes, it is possible that they could be syndemic factors working together to worsen HIV-related health. As such, we qualitatively explored the circumstances in which food insecurity and IPV co-occur and the mechanisms through which their convergence influences HIV risk and treatment behaviors among women living with and at risk for HIV in the U.S.

## Methods

### Research design and setting

We conducted a qualitative study nested within the Women’s Interagency HIV Study (WIHS), now part of the Multicenter AIDS Cohort Study-WIHS Combined Cohort Study (MWCCS). WIHS was a multicenter prospective cohort study of women living with HIV and demographically similar HIV-negative controls with a history of sexually transmitted infections or behavioral or demographic characteristics that increased their risk of acquiring HIV, in nine sites across the U.S. [57]. Survey, clinical, and biological data were collected semi-annually from WIHS participants. In this qualitative study, we aimed to explore how experiences of IPV and food insecurity intersect and influence HIV risk and treatment behaviors among women enrolled in the San Francisco, California and Atlanta, Georgia WIHS sites. Women at the San Francisco site lived in the city itself and the urban and suburban settings of the greater Bay Area. Women at the Atlanta site lived in the city and in the surrounding suburban and rural areas. We refer to these sites as Northern California and Georgia to account for these geographic differences within each site. These two sites were chosen due to their geographic diversity and differences in social welfare provision; despite high poverty rates in both locations, California has above average per capita welfare spending, while Georgia has one of the lowest per capita welfare spending in the country [58]. The large number of WIHS women experiencing both food insecurity and IPV at these sites also made our ability to recruit the sample more feasible.

### Sampling and recruitment

We recruited women from the Northern California and Georgia WIHS sites from January through March 2019 who met our inclusion criteria of reporting: 1) physical, sexual or emotional violence from an intimate partner in the past year at their most recent WIHS visit; and 2) marginal, low or very low food security, as defined by the Household Food Security Survey Module [3, 59], at or before their most recent WIHS visit. Participants were also purposively sampled so that half were living with HIV. This approach allowed us to explore whether experiences of IPV and food insecurity differed by HIV status and how their intersection may influence HIV risk behaviors as well as engagement in HIV care and treatment. WIHS staff from each site contacted eligible cohort members over the phone to assess their interest in participating in the study. If interested, the study interviewer contacted them to schedule an interview.

### Data collection

A semi-structured interview guide was developed by the first author, with input from SDW, AAC and PCT. The guide included broad, open-ended questions that asked about experiences with violence and food insecurity and perspectives on how such experiences influence HIV risk and treatment behaviors. For example, to explore experiences of food insecurity, participants were asked to describe what they ate on a daily basis, their perception of the quality of food they ate, and any challenges they faced in accessing the amounts and types of food they desired. The guide also included questions asking about the circumstances under which women experienced physical, sexual and emotional violence from partners. The guide included probes to explore the ways food insecurity and IPV might overlap, such as experiencing violence in the context of arguments over lack of food or feeling unable to leave an abusive relationship due to reliance on one’s partner for food. Finally, the guide included questions designed to explore how experiences with food insecurity and violence influenced women’s emotional well-being and their ability to manage their health including using condoms, attending clinic appointments and taking medication, including ART. Participants were also asked about how experiences of food insecurity and violence might influence their engagement in other HIV risk behaviors including condomless sex and transactional sex. Questions in the guide were informed by a literature review and our prior research on these topics [13, 45, 54, 60, 61].

A trained female Master’s-level researcher with qualitative interviewing experience conducted the interviews in English in a private office at the local WIHS site. Interviews lasted between 60–120 minutes each and were audio-recorded and transcribed verbatim. Participants were assigned unique identification numbers and compensated $55 at the end of each interview for their time.

### Ethics

This study received human subjects research approval from the Institutional Review Board of the University of California San Francisco (UCSF) (site lead for the Northern California WIHS), and by the WIHS Executive Committee. Emory University (site lead for the Georgia WIHS) waived approval because the Georgia data was collected by UCSF researchers. All participants provided written informed consent before starting the interview.

### Data analysis

Data were analyzed using thematic content analysis methodology [62] following an inductive-deductive approach [63]. An initial codebook was developed by AML and JS, based on *a priori* codes informed by prior research. The codebook was iteratively revised during the analytic process by adding new inductive codes to reflect emergent themes identified during transcript review. The researchers met regularly to discuss emergent themes and codes and their application until they reached consensus around a final codebook. JS coded the transcripts, using memos to elaborate upon the codes and their application. AML reviewed the codes of approximately one-quarter of the transcripts, and JS and AML discussed disagreements in coding and came to a consensus. JS single coded the remainder of the transcripts. Once all transcripts were coded, AML and JMZ analyzed the data. Coded segments related to our research question, describing how violence and food insecurity intersect and influence HIV risk and treatment behaviors were iteratively reviewed. Key themes were developed and refined with descriptions of common and divergent experiences and perspectives and illustrative quotes representing both common and divergent perspectives. The key themes were reviewed by authors, with discrepancies resolved through discussion and consensus. These results are summarized below, with quotes anonymously identified by pseudonym, site, HIV status, race/ethnicity, and age.

## Results

**Fig 1:**
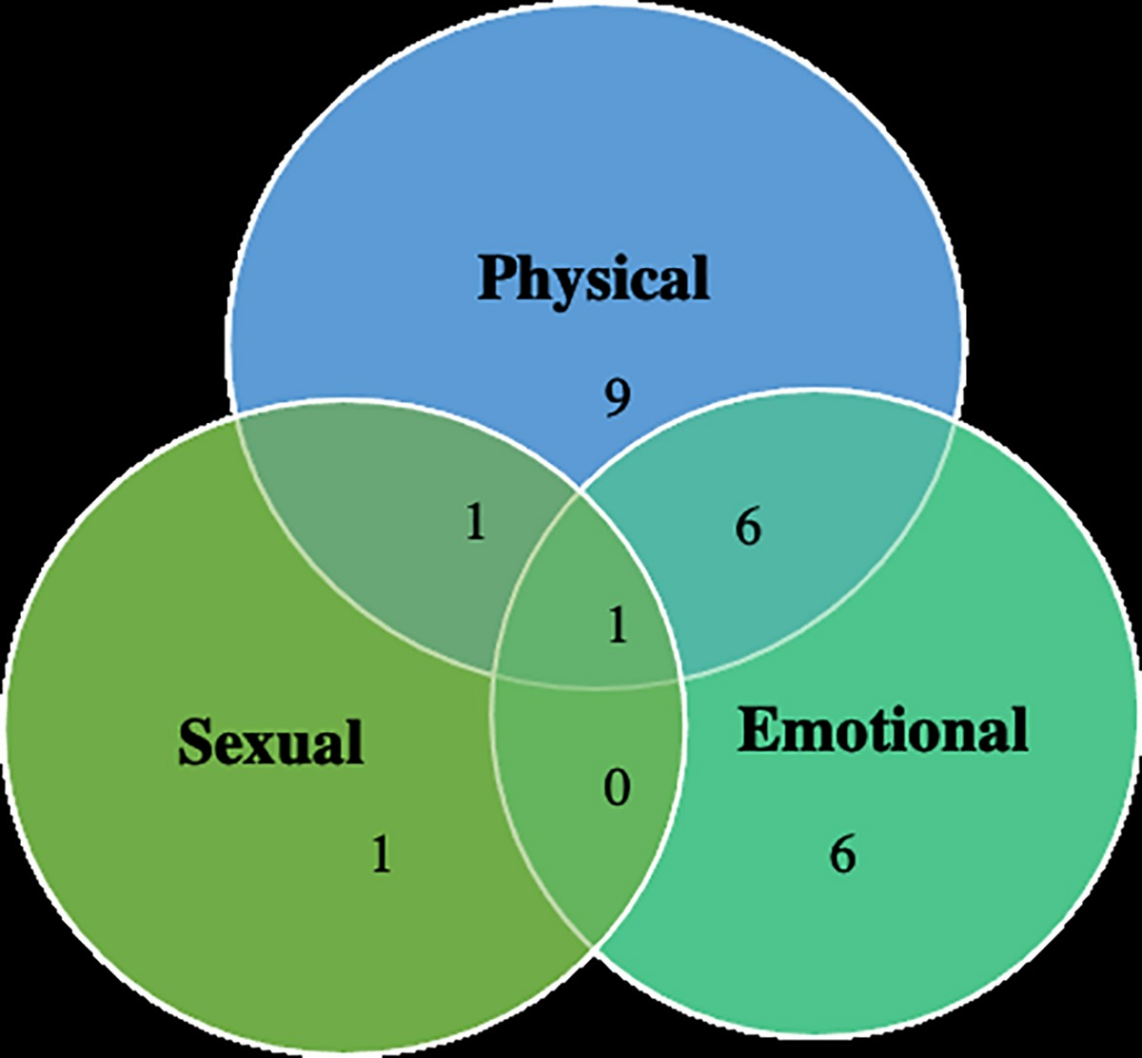
Forms of violence reported (n = 24).

We interviewed 12 participants from the Northern California site (n = 7 HIV-positive; n = 5 HIV-negative) and 12 participants from the Georgia site (n = 5 HIV-positive; n = 7 HIV-negative). All women were cisgender, and the majority reported very low food security, meaning that they experienced disruptions in their eating patterns or reduced food intake because they lacked the resources to access food [64] (Table 1). The most common forms of violence were physical violence, emotional violence, and concurrent experiences of physical and emotional violence (Fig 1). Nearly half the women described experiences of child abuse (n = 10). The majority of participants reported relying on social safety net programs such as Supplemental Security Income, Social Security Disability, and Supplemental Nutrition Assistance Program (SNAP) (i.e., food stamps) to access income and food. All participants described these services as insufficient to meet their daily needs. Half of the sample were living with HIV, and most participants revealed that they had other chronic conditions such as diabetes mellitus and hypertension.

**Table 1:** Participant characteristics (n = 24).

Characteristic	n/mean (range)
Age, mean (range)	49 (29–71)
Past year food security status ^a^	
Very low food security	17
Low food security	3
Marginal food security	4
Any intimate partner violence in past year ^b^	24
Race/Ethnicity	
Black/African American	16
Non-Hispanic White	4
Other	4
Living with HIV	12
Ever experienced unstable housing ^c^	15
Ever used drugs	15
Ever had mental health conditions	18
Experienced child abuse^d^	10

^a^Food security status determined by the Household Food Security Survey Module.

^b^IPV includes physical, sexual, and emotional violence.

^c^Unstable housing includes experiencing homelessness or living in overcrowded conditions.

^d^Child abuse includes neglect and physical, sexual, and emotional abuse.

A dominant theme that emerged at both sites was one of co-occurring and mutually reinforcing experiences of violence, food insecurity, poor mental health, and substance use. Specifically, most participants described how intersecting experiences of violence and food insecurity caused or exacerbated poor mental health outcomes and spurred their use of illicit substances as a coping strategy. At the same time, poor mental health and substance use amplified risks for violence and food insecurity. The converging experiences of violence, food insecurity, poor mental health and substance use were described to increase HIV risk behaviors and undermine optimal health management, including HIV care and treatment among WLHIV. Findings did not differ by site.

### Intersecting experiences of violence and food insecurity/material deprivation

Many women described how, in the context of food insecurity and other material need insecurities (e.g., housing, income), they often needed to rely on intimate partners or strangers to meet their daily living needs. Women noted how this reliance created unequal power dynamics that left them vulnerable to abuse. Some women said men coerced them to engage in unwanted sexual acts in exchange for food, both in the context of intimate partnerships and sex work.

We did have conflict over food sometimes because he would bring food into my house and then if I don’t have sex with him or something he would want to take it back. So, that happened several times that he would get mad and say oh, I’m taking my food. (Harriet, 40 years old, African American, HIV-negative, Georgia)

Women who depended on intimate partners to meet their material needs also described feeling unable to leave abusive relationships, particularly if they wanted to ensure consistent access to resources for their children.

We’d get just in a bad situation where we think the guy’s going to help us out and help us with food and our children and stuff. … And instead it turns into an abusive relationship—some women just…let it happen because these day and time it’s hard to…get a meal. (Doris, 55 years old, African American, HIV-positive, Northern California)

Some women revealed that their experiences with food insecurity began after leaving an abusive partner whom they relied on to access food. For example, Barbara, a 53-year-old HIV-negative white woman from Northern California described how leaving her abusive partner after a particularly violent encounter sent her down a path of food insecurity and homelessness: “I never went back after that. So that’s what started my life like being really homeless and hungry and everything”.

Another theme was stress over lack of food as a cause of conflict and violence in relationships. For example, several participants described conflict arising when their partners would eat most or all the food available for the week, leaving nothing for the rest of the family.

It kind of pissed me off ’cause the way I had to calculate everything… it’s enough just for me and my kids. Then when my baby daddy back in the day was staying with me…he’ll try to eat up everything… But he didn’t buy the food. And then if he get mad at me and shit, he gonna take my shit out the freezer and shit and sell it, and he ain’t thinking about the kids. (Candice, 52 years old, African American, HIV-positive, Northern California)

Candice revealed that this behavior caused her to fight with her partner, in some cases resulting in severe physical violence. After one such conflict, she described her partner beating her to such an extent that “he tried to kill [her]”.

### Violence, food insecurity, poor mental health outcomes and substance use: A vicious cycle

The majority of women described how co-occurring experiences of food insecurity and violence contributed to feelings of low self-esteem, psychological trauma, and depression. To cope, many women described using substances to “get rid of the pain”. At the same time, poor mental health and substance use further perpetuated food insecurity and violence.

#### Mental health experiences

The majority of women noted how experiences of violence and food insecurity were associated with feelings of low self-worth and “failure”. Sofia, a 51-year-old woman living with HIV in Georgia, shared that, “you’ll be like, man, nobody care, give a shit about me”. When reflecting on how experiences with food insecurity made her feel, another woman said:

It has made me feel unworthy, you know, that I can’t get food. It makes me feel less of a person…I’m not worthy of getting the basic stuff that I need. (Brianna, 49 years old, African American, HIV-negative, Northern California)

The experiences of food insecurity, violence, and substance use caused women emotional pain and long-term mental duress that they struggled to get past. For example, Barbara, who became homeless and food insecure after she left her abusive partner, and then had to exchange sex for food while living on the streets, described how her experiences affected her mental health in the following way:

It’s taken a toll on my psyche, period, because sometimes I—and this is the PTSD. I can smell something or go past a certain place, and I relive everything. … It just keeps me on pins and needles, and I constantly relive it over and over and over again…It has taken a toll on my psyche. It makes me paranoid. (Barbara, 53 years old, White, HIV-negative, Northern California)

Some women who experienced severe physical discomfort from extreme hunger also remembered the feeling vividly even years later, causing them ongoing distress.

Several women described that the poor mental health effects noted above often led them into bouts of what they described as depression in which they withdrew from life, did not see other people, and stayed in bed or slept all day. When asked about how difficulties accessing food made her feel, one woman with very low food security said:

…it deepens my depression, definitely, and I already have some days where I really don’t even want to get up and get out of bed and do anything. And it definitely doesn’t help my energy or anything like that not to be able to go in the kitchen and fix a nutritious meal…Some days I might just lay there unless I have something specific to do. I’ll sleep most of the day off. So I’m not thinking about food or whatever else. (Serena, 37 years old, African American, HIV-positive, Georgia)

In extreme situations, some women felt driven to suicidal thoughts or attempts. Another woman with marginal food security and a history of violence and substance use said:

…I tried to commit suicide a few times. … [B]ecause I didn’t want to do anymore drugs. I was tired of being on the streets, I was tired of being abused, I was tired of being homeless, I was just tired, you know, and I felt like if I would just take some pills and just sleep and never wake up. (Dorothy, 49 years old, African American, HIV-negative, Georgia)

#### Substance use as a coping mechanism

Many women spoke of using drugs and alcohol as a way to cope with the mental health effects of food insecurity and violence. Shirley, who experienced physical, emotional, and sexual violence from intimate partners and engaged in sex work and begging to procure food while living on the streets, reflected on the role of drugs in dealing with trauma from these experiences:

[M]ental illness, which is all that trauma…and drugs go hand-in-hand. They go hand-in-hand because mental illness most of the times, well, for me, is what caused me to use dope…I wanted to suppress all the shit that had happened, and I didn’t know no other coping mechanism but to get high. (Shirley, 57 years old, African American, HIV-positive, Northern California)

Dorothy described how she used drugs to cope with her experiences of abuse and her feelings of low self-worth after exchanging sex for food and other resources:

…a lot of times, I dealt with the drugs because I didn’t want to deal… that was my…relief. You know, like, I didn’t want to feel no pain, I didn’t want to feel no hurt, I didn’t want to feel…the feelings that I was feeling about myself… And sometimes… sucking a man’s dick outside, around the corner in the bushes, you know, just like bent over, it was just degrading where I took myself. (Dorothy, 49 years old, African American, HIV-negative, Georgia)

#### Perpetuating the cycle of food insecurity and violence

Poor mental health outcomes and substance use were described to perpetuate the cycle of food insecurity and violence. Some women said that the mental health impacts of abuse and food insecurity rendered them emotionally vulnerable to abusive and controlling men. A woman from Georgia with a life-long history of abuse who experienced very low food security said, “You know the parts of me that felt unloved and … low self-esteem allowed me to be in abusive situations…” (Molly, 29 years old, White, HIV-negative, Georgia).

Several women also described how substance use placed them at risk of violence and exacerbated their food insecurity. Some women noted how they or their partners would use all of their money on drugs or alcohol and would not have any money left to purchase food, leading to conflict.

There was a time where food wasn’t available because of… bad habits like drugs… and it became an argument about what happened to the food?…Somebody exchanged food for money to get [drugs] instead of what the family wanted or needed (Margaret, 44 years old, African American, HIV-negative, Northern California)

Conflict over lack of food could become particularly violent in the context of substance use.

One time I was hungry and both of us was [high], but I put the blame on him and it caused an argument…We had violence a couple of times…We fought one time when we was clean, but it wasn’t as violent as it was when we was on drugs. (Harriet, African American, 40 years old, HIV-negative, Georgia)

### Effects on health management including HIV risk and engagement in HIV care and treatment

Women described how intersecting experiences of food insecurity, violence, poor mental health and substance use increased their HIV acquisition risk and undermined optimal health management including HIV care and treatment.

#### Increased HIV risk

Women described experiencing sexual violence when they exchanged sex for food and/or money, thereby increasing their risk for HIV acquisition. Substance use was noted to amplify these risks by increasing the frequency of sexual exchange for drugs or drug money and by creating dangerous situations in which sexual and physical violence or coercion could occur. One woman with a history of exchanging sex for food and money said:

I think people that’s got drugs and alcohol, any kind of that substance… are more capable of getting infections like that [HIV] because they’re constantly kind of like numb…So I think that it made me more easy to get caught up in situations. And especially the guys, because they feel real loose and they will rape you and bunches take turns (Margaret, 44 years old, African American, HIV-negative, Northern California)

Poor mental health outcomes resulting from the intersection of abuse and food insecurity were described to increase the likelihood of future substance use and of risky drug or sexual behaviors due to low self-regard or “giving up”. As noted in the following quote, injection drug use carried its own risk of HIV acquisition.

I used to get so messed up behind these men. Let me tell you what I did. This is how I got it (HIV). I went and shot dope with somebody I knew to have full-blown AIDS, and I didn’t care. I wanted to die because of how this man was treating me and kicking me around. I didn’t know what to do. (Shirley, 57 years old, African American, HIV-positive, Northern California)

As described earlier, poor mental health status also made it more difficult for some women to leave abusive relationships, maintaining the cycle of violence and/or food insecurity that increased HIV acquisition risk.

#### Medication non-adherence

Several women shared that they missed their medication doses during periods of extreme depression when they didn’t leave their house (or even their beds). Low appetite and desire for food during these periods also undermined their ability to take certain medications that required food. The stress and emotional upheaval of food insecurity and especially of violence also made it difficult for women to take their medication as prescribed. Substance use further undermined medication adherence and increased the chances of an empty stomach since women often described forgoing food during periods of intense substance use. These factors are captured in the following quote:

…sometimes if I know I haven’t eaten, I might not take a certain medication…If I’m not eating, I don’t want my stomach to feel really bad…sometimes if I’m going through those violent situations, to block all that or to counteract that, I might start using. And then if I’m using, I’m definitely not eating or taking the medication. (Serena, 37 years old, African American, HIV-positive, Georgia)

Another woman living with HIV described how emotional violence, poor mental health and substance use prevented her from taking her ART.

They get to call me a bunch of names. We get to fighting. I go get high, and guess what? [I don’t take my medication]… My viral load and everything. It affected me to the point where my doctor told me this is my last regimen. She says she ain’t got no more combinations. This is my last one. (Shirley, 57 years, African American, HIV-positive, Northern California)

While ART non-adherence was more frequently described, many study participants also struggled with medication adherence for treatment of pre-existing mental and chronic health conditions (e.g., clinical depression, bipolar disorder, diabetes, hypertension). In the following quote, a woman describes how her lack of food and poor mental health stemming from experiences of violence leads her to withdraw and miss her medications for arthritis, hypertension, depression, anxiety and other ailments.

If I don’t eat well I have less energy. I’ll get a headache. My stomach starts bothering me…It just affects me wanting to get up and take care of myself sometimes. I just want sometimes to just lay down and just sleep all day or something like that…I take ten pills a day, so I have to put something into my system. (Brianna, 49 years old, African American, HIV-negative, Northern California)

#### Missed clinic appointments

Finally, women described being more likely to miss clinic appointments when they isolated at home during periods of depression due to violence and food insecurity. Many remarked that if they were actively using substances or hung over after a period of intense use, they were more likely to skip an appointment in order to hide this from the doctor.

Well, my ex-husband, we’d get in a fight or something, and he’d get to call me a bunch of maggots and all that kind of stuff. Then I want to go get high, and if I get high, I’m not going to the doctor. I’m not going. (Shirley, 57 years old, African American, HIV-positive, Northern California)

Women also avoided doctors out of embarrassment or shame when they had physical signs of abuse. Sofia, a 51-year-old woman living with HIV from Atlanta, said, “I couldn’t even go to the doctor ’cause I had black eyes. So, that’s—it’s very embarrassing”.

Other participants described how in the context of food insecurity and hunger, they chose to skip clinic visits to access food at food banks, or to save the money they would have used on transport to the clinic to purchase food.

And even with my doctor’s appointment if it’s like in the…morning time, I have difficulty getting there if I haven’t eaten anything… So, I’m hungry and I have to choose and pick what’s more important. So, I choose food. (Brianna, 49 years, African American, HIV-negative, Northern California)

Women experiencing extreme hunger described sometimes lacking the energy to physically get to clinic appointments. For example, when asked how experiences of food insecurity influenced her ability to attend clinic visits, Beth, a 71-year-old African American woman living with HIV from Northern California experiencing very low food security said that she sometimes felt “too weak” from lack of food that she would stay in bed and miss her appointments.

## Discussion

In this study, we aimed to explore how experiences of food insecurity and IPV intersect and shape HIV risk and treatment behaviors among women living with and at risk for HIV in two geographic areas of the U.S. Our findings revealed that converging experiences of food insecurity and violence led to or exacerbated poor mental health conditions, and the majority noted turning to substances to help them cope. Poor mental health outcomes and substance use then continued to perpetuate the cycle of food insecurity and violence. Ultimately, the co-occurring and mutually reinforcing interactions between food insecurity, IPV, poor mental health and substance use contributed to worse health outcomes by increasing HIV risk behaviors and undermining engagement in HIV care and treatment.

In line with syndemic theory, all participants in this study described lives shaped by structural disadvantage, including unemployment, poverty, unstable housing, food insecurity, poor overall health status, and life-long histories of violence. Given that the majority of participants in this study were African American, it is also important to acknowledge that the socio-structural context described by participants is largely a product of structural racism- policies implemented throughout U.S. history that have systematically denied African Americans equal access to social, economic and health resources [65, 66]. Our findings suggest that this constellation of structural disadvantage gives rise to the clustering of epidemics of IPV, food insecurity, poor mental health, and substance use among this population of low-income, largely African American women. The synergistic effects of these four epidemics is perhaps best illustrated by the study participant Shirley, who described acquiring HIV by knowingly injecting heroin with someone she knew to have AIDS because of her suicidal thoughts driven by intersecting experiences of food insecurity and violence. Her use of substances to cope with her poor mental health condition led her to consistently miss ART doses and fail HIV treatment so many times that she is currently on the last line of HIV treatment available.

This syndemic and its negative impact on health outcomes may be even more pervasive during the COVID-19 pandemic. Emerging evidence suggests that rates of food insecurity, IPV, poor mental health and substance use have risen substantially since the onset of the COVID-19 pandemic and associated social distancing policies [67–69]. Thus, it is possible that these factors may also synergistically intersect at higher rates among vulnerable women, such as those included in the present study, to exacerbate negative health outcomes, including HIV-related morbidity and mortality. Indeed, recent evidence suggests that engagement in HIV care and treatment has declined among people living with HIV since the onset of the social distancing policies [70].

Our findings build on past research that has documented a relationship between food insecurity and IPV, and their independent effects on substance use, mental health, and HIV outcomes. Food insecurity and IPV are independently associated with poor mental and physical health outcomes including depression, anxiety, trauma, low self-esteem, and suicidal ideation [60, 71–74], substance use [74, 75], HIV risk behavior [8–11] and poor engagement in HIV care and treatment [12–16]. Substance use and poor mental health, in turn, are also associated with each other [76, 77] and independently with HIV risk behaviors [78–80] and worse HIV outcomes [81–83]. Findings from the present study extend this past research to suggest that IPV, food insecurity, poor mental health and substance use co-occur and synergistically interact to exacerbate HIV risk behaviors and worsen engagement in HIV care and treatment.

Our results also expand upon the extensive research into the SAVA syndemic [35, 49], which has demonstrated SAVA’s impact on increased HIV risk behaviors, poor mental health outcomes, and worse HIV care and treatment outcomes among U.S. women [35, 84, 85]. Specifically, our results highlight that food insecurity and poor mental health conditions also synergistically interact with violence, substance use, and HIV/AIDS, leading to increased HIV risk behaviors and worse engagement in HIV care and treatment. Findings from this study highlight the myriad ways in which these factors interact to impact HIV risk and treatment behaviors that quantitative studies have not uncovered to date and suggest that simple associations between two variables may be insufficient to understand the complex lived experiences of women living with or at risk for HIV. Future quantitative studies should employ a syndemic approach to confirm the intersections uncovered in this paper.

### Strengths and limitations

Our study was limited to WIHS participants, which includes cisgender and largely heterosexual women who have participated in the cohort for several decades, and thus may not be generalizable to all women living with or at risk of HIV in the U.S. Our findings are strengthened, however, by the use of two distinct geographic locations that may offer important insights into common patterns in experiences, with potential implications for the broader U.S. context. We have provided detailed descriptions of our study context, methods, and sample to assist readers in determining whether and how our findings apply to other contexts. Future studies should continue to explore the intersection of food insecurity and IPV among other important populations, including undocumented immigrants, who face citizenship-related barriers to accessing government social services such as food aid [86] and violence support services [87]. Research is also needed to explore this syndemic among queer individuals, particularly transgender (‘trans’) women, who are disproportionately affected by HIV and IPV [88, 89] and experience extremely high rates of food insecurity, poor mental health outcomes and substance use due to stigma and discrimination related to their gender identity/ expression [90–92]. The sensitive and retrospective nature of some of our interview questions may have made our findings less representative of and faithful to the participants’ lived experience [93]. To mitigate this and to increase participant comfort, we utilized a sex-matched interviewer trained in qualitative and trauma-informed methods, including neutral probing and strategies to address participant discomfort, and conducted the interviews at WIHS offices already familiar to participants. Nevertheless, the similarity and frequency of themes we found among participants both living with and at risk for HIV across both study sites underscores the strength of our findings.

### Research implications and conclusions

To our knowledge, this is one of the first studies to describe a syndemic of food insecurity, IPV, poor mental health and substance use and its negative influence on HIV prevention and treatment behaviors. Quantitative research is needed to understand how common this syndemic is and to elucidate the synergistic effects of these multiple factors on HIV outcomes. It will also be important for research to examine the social and structural drivers of this syndemic and how policies, such those designed to increase food access through the Biden-Harris Administration’s newly enacted American Rescue Plan, [94] can address this syndemic and improve HIV prevention and treatment outcomes. Further, while we focused on HIV as the core health issue among these women, our findings also suggest that HIV is just one of many health issues affected by the syndemic of food insecurity, IPV, poor mental health and substance use. As WLHIV age and develop other comorbidities- a rising problem in the U.S. [95], future research should explore how syndemics like the one presented in this paper can impact long-term health and wellbeing.

Findings from this study also point to the need for multi-level and trauma-informed interventions that address the social and structural context in addition to individual-level health behaviors [50]. For example, interventions could include some of the following components: providing food and economic support, conflict resolution skills, mental health and substance use support, and support for HIV prevention and engagement in HIV care and treatment. Such an approach could also include policies that increase and expand eligibility to SNAP, which has been shown to reduce food insecurity and poverty and improve health outcomes, including mental health and medication adherence [96]. A trauma-informed approach may also be particularly useful to address this syndemic. Trauma-informed care provides a framework to increase providers’ understanding that exposure to trauma, such as IPV and food insecurity, impacts all aspects of survivors’ lives and shapes coping strategies, such as substance use and health care avoidance [97]. It also enhances providers’ ability to recognize the signs and symptoms of trauma and guides their responses to include support provision and helping women develop a safety plan when they feel they are at risk of violence. Trauma-informed care has been shown to improve mental health and substance use outcomes and has been identified as a promising approach to improve HIV prevention and treatment outcomes [98–100]. Ultimately, our findings suggest that addressing the syndemic of food insecurity, IPV, poor mental health and substance use among women living with and at risk for HIV in the U.S. may be critical to improving HIV prevention and treatment outcomes among this population.

## References

[1] Breiding MJ, Chen J., Black M.C. Intimate partner violence in the United States—2010. Atlanta, GA: National Center for Injury Prevention and Control, Centers for Disease Control and Prevention; 2014.

[2] Coleman-Jensen A, Rabbitt MP, Gregory CA, Singh A. Household Food Insecurity in the United States in 2018, ERR-270. 2019.

[3] National Research Council. Food insecurity and hunger in the United States: An assessment of the measure. Panel to review the U.S. Department of Agriculture’s measurement of food insecurity and hunger. Washington, D.C.; 2006.

[4] Spinelli MA, Frongillo EA, Sheira LA, Palar K, Tien PC, Wilson T, et al. Food Insecurity is Associated with Poor HIV Outcomes Among Women in the United States. AIDS Behav. 2017. 10.1007/s10461-017-1968-2 29119474 PMC5824627

[5] Smith SG, Zhang X., Basile K.C., Merrick M.T., Wang J., Kresnow M., et al. The National Intimate Partner and Sexual Violence Survey: 2015 Data Brief- Updated Release Atlanta, GA: National Center for Injury Prevention and Control, Centers for Disease Control and Prevention; 2018 [Available from: https://www.cdc.gov/violenceprevention/pdf/2015data-brief508.pdf.

[6] Machtinger EL, Wilson TC, Haberer JE, Weiss DS. Psychological trauma and PTSD in HIV-positive women: a meta-analysis. AIDS Behav. 2012;16(8):2091–100. 10.1007/s10461-011-0127-4 22249954

[7] Breiding MJ, Basile K.C., Smith S.G., Black M.C., Mahendra R.R. Intimate Partner Violence Surveillance: Uniform Definitions and Recommended Data Elements, Version 2.0 Atlanta, GA: National Center for Injury Prevention and Control, Centers for Disease Control and Prevention; 2015 [Available from: https://www.cdc.gov/violenceprevention/pdf/ipv/intimatepartnerviolence.pdf.

[8] Teitelman AM, Ratcliffe SJ, Morales-Aleman MM, Sullivan CM. Sexual relationship power, intimate partner violence, and condom use among minority urban girls. Journal of Interpersonal Violence. 2008;23(12):1694–712. 10.1177/0886260508314331 18349344 PMC3677854

[9] Lang DL, Salazar LF, Wingood GM, DiClemente RJ, Mikhail I. Associations between recent gender-based violence and pregnancy, sexually transmitted infections, condom use practices, and negotiation of sexual practices among HIV-positive women. J Acquir Immune Defic Syndr. 2007;46(2):216–21. 10.1097/QAI.0b013e31814d4dad 17693895

[10] Weiser SD, Leiter K, Bangsberg DR, Butler LM, Percy-de Korte F, Hlanze Z, et al. Food insufficiency is associated with high-risk sexual behavior among women in Botswana and Swaziland. PLoS Med. 2007;4(10):1589–97; discussion 98. 10.1371/journal.pmed.0040260 17958460 PMC2039764

[11] Tsai AC, Hung KJ, Weiser SD. Is food insecurity associated with HIV risk? Cross-sectional evidence from sexually active women in Brazil. PLoS Med. 2012;9(4):e1001203. 10.1371/journal.pmed.1001203 22505852 PMC3323512

[12] Weiser SD, Palar K, Frongillo EA, Tsai AC, Kumbakumba E, Depee S, et al. Longitudinal assessment of associations between food insecurity, antiretroviral adherence and HIV treatment outcomes in rural Uganda. AIDS. 2014;28(1):115–20. 10.1097/01.aids.0000433238.93986.35 23939234 PMC4629837

[13] Leddy AM, Weiss E, Yam E, Pulerwitz J. Gender-based violence and engagement in biomedical HIV prevention, care and treatment: a scoping review. BMC Public Health. 2019;19(1):897. 10.1186/s12889-019-7192-4 31286914 PMC6615289

[14] Young S, Wheeler AC, McCoy SI, Weiser SD. A review of the role of food insecurity in adherence to care and treatment among adult and pediatric populations living with HIV and AIDS. AIDS and behavior. 2014;18 Suppl 5:S505–15. 23842717 10.1007/s10461-013-0547-4 PMC3888651

[15] Weiser SD, Fernandes KA, Brandson EK, Lima VD, Anema A, Bangsberg DR, et al. The association between food insecurity and mortality among HIV-infected individuals on HAART. J Acquir Immune Defic Syndr. 2009;52(3):342–9. 10.1097/QAI.0b013e3181b627c2 19675463 PMC3740738

[16] Closson K, McLinden T, Parry R, Lee M, Gibbs A, Kibel M, et al. Severe intimate partner violence is associated with all-cause mortality among women living with HIV. AIDS. 2020;34(10):1549–58. 10.1097/QAD.0000000000002581 32675565

[17] Dunkle KL, Decker MR. Gender-based violence and HIV: reviewing the evidence for links and causal pathways in the general population and high-risk groups. American journal of reproductive immunology (New York, NY: 1989). 2013;69 Suppl 1:20–6. 10.1111/aji.12039 23216606

[18] Wang EA, McGinnis KA, Fiellin DA, Goulet JL, Bryant K, Gibert CL, et al. Food insecurity is associated with poor virologic response among HIV-infected patients receiving antiretroviral medications. J Gen Intern Med. 2011;26(9):1012–8. 10.1007/s11606-011-1723-8 21573882 PMC3157515

[19] Siemieniuk RA, Krentz HB, Miller P, Woodman K, Ko K, Gill MJ. The clinical implications of high rates of intimate partner violence against HIV-positive women. Journal of acquired immune deficiency syndromes (1999). 2013;64(1):32–8. 10.1097/QAI.0b013e31829bb007 23714742

[20] Lichtenstein B. Domestic violence in barriers to health care for HIV-positive women. AIDS Patient Care and STDs. 2006;20(2):122–32. 10.1089/apc.2006.20.122 16475893

[21] Kosia A, Kakoko D, Semakafu AM, Nyamhanga T, Frumence G. Intimate partner violence and challenges facing women living with HIV/AIDS in accessing antiretroviral treatment at Singida Regional Hospital, central Tanzania. Global health action. 2016;9:32307. 10.3402/gha.v9.32307 27987296 PMC5161793

[22] Watt MH, Dennis AC, Choi KW, Ciya N, Joska JA, Robertson C, et al. Impact of Sexual Trauma on HIV Care Engagement: Perspectives of Female Patients with Trauma Histories in Cape Town, South Africa. AIDS and behavior. 2016. 10.1007/s10461-016-1617-1 PMC5438301 27866288

[23] Hatcher AM, Turan JM, Leslie HH, Kanya LW, Kwena Z, Johnson MO, et al. Predictors of linkage to care following community-based HIV counseling and testing in rural Kenya. AIDS and behavior. 2012;16(5):1295–307. 10.1007/s10461-011-0065-1 22020756 PMC3590795

[24] Lopez EJ, Jones DL, Villar-Loubet OM, Arheart KL, Weiss SM. Violence, coping, and consistent medication adherence in HIV-positive couples. AIDS Education and Prevention: Official Publication of the International Society for AIDS Education. 2010;22(1):61–8. 10.1521/aeap.2010.22.1.61 PMC3734535 20166788

[25] Trimble DD, Nava A, McFarlane J. Intimate partner violence and antiretroviral adherence among women receiving care in an urban Southeastern Texas HIV clinic. Journal of the Association of Nurses in AIDS Care. 2013;24(4):331–40. 10.1016/j.jana.2013.02.006 23790276

[26] Hampanda KM. Intimate partner violence and HIV-positive women’s non-adherence to antiretroviral medication for the purpose of prevention of mother-to-child transmission in Lusaka, Zambia. Social science & medicine (1982). 2016;153:123–30. 10.1016/j.socscimed.2016.02.011 26896876 PMC4788551

[27] Kidman R, Violari A. Dating violence against HIV-infected youth in South Africa: Associations with sexual risk behavior, medication adherence, and mental health. J Acquir Immune Defic Syndr. 2017. 10.1097/QAI.0000000000001569 PMC5720896 29040165

[28] Mepham S, Zondi Z, Mbuyazi A, Mkhwanazi N, Newell ML. Challenges in PMTCT antiretroviral adherence in northern KwaZulu-Natal, South Africa. AIDS Care. 2011;23(6):741–7. 10.1080/09540121.2010.516341 21293987

[29] Human Rights Watch. Hidden in the Mealie Meal: Gender-based abuses and women’s HIV treatment in Zambia. Washington, DC: Human Rights Watch; 2007. Contract No.: Report.

[30] Conroy A, Leddy A, Johnson M, Ngubane T, van Rooyen H, Darbes L. ’I told her this is your life’: relationship dynamics, partner support and adherence to antiretroviral therapy among South African couples. Culture, health & sexuality. 2017:1–15. 10.1080/13691058.2017.1309460 28398134 PMC5626574

[31] Hatcher AM, Stockl H, Christofides N, Woollett N, Pallitto CC, Garcia-Moreno C, et al. Mechanisms linking intimate partner violence and prevention of mother-to-child transmission of HIV: A qualitative study in South Africa. Social science & medicine (1982). 2016;168:130–9. 10.1016/j.socscimed.2016.09.013 27643847

[32] Hatcher AM, Woollett N, Pallitto CC, Mokoatle K, Stockl H, MacPhail C, et al. Bidirectional links between HIV and intimate partner violence in pregnancy: implications for prevention of mother-to-child transmission. Journal of the International AIDS Society. 2014;17:19233. 10.7448/IAS.17.1.19233 25371218 PMC4220001

[33] Maeri I, El Ayadi A, Getahun M, Charlebois E, Akatukwasa C, Tumwebaze D, et al. "How can I tell?" Consequences of HIV status disclosure among couples in eastern African communities in the context of an ongoing HIV "test-and-treat" trial. AIDS Care. 2016;28 Suppl 3:59–66. 10.1080/09540121.2016.1168917 27421052 PMC5751752

[34] Conroy AA, Leddy AM, Darbes LA, Neilands TB, Mkandawire J, Stephenson R. Bidirectional Violence Is Associated with Poor Engagement in HIV Care and Treatment in Malawian Couples. J Interpers Violence. 2020:886260520959632. 10.1177/0886260520959632 32946327 PMC7969480

[35] Sullivan KA, Messer LC, Quinlivan EB. Substance abuse, violence, and HIV/AIDS (SAVA) syndemic effects on viral suppression among HIV positive women of color. AIDS Patient Care and STDs. 2015;29 Suppl 1:S42–8. 25397666 10.1089/apc.2014.0278 PMC4283071

[36] Rose RC, House AS, Stepleman LM. Intimate partner violence and its effects on the health of African American HIV-Positive women. Psychological Trauma: theory, Research, Practice, and Policy. 2010;2(4):311–7.

[37] Espino SR, Fletcher J, Gonzalez M, Precht A, Xavier J, Matoff-Stepp S. Violence screening and viral load suppression among HIV-positive women of color. AIDS Patient Care and STDs. 2015;29 Suppl 1:S36–41. 10.1089/apc.2014.0275 25561308 PMC4283058

[38] Ricks JL, Cochran SD, Arah OA, Williams JK, Seeman TE. Food insecurity and intimate partner violence against women: results from the California Women’s Health Survey. Public Health Nutr. 2016;19(5):914–23. 10.1017/S1368980015001986 26096652 PMC6884316

[39] Melchior M, Caspi A, Howard LM, Ambler AP, Bolton H, Mountain N, et al. Mental health context of food insecurity: a representative cohort of families with young children. Pediatrics. 2009;124(4):e564–72. 10.1542/peds.2009-0583 19786424 PMC4231784

[40] Montgomery BE, Rompalo A, Hughes J, Wang J, Haley D, Soto-Torres L, et al. Violence Against Women in Selected Areas of the United States. Am J Public Health. 2015;105(10):2156–66. 10.2105/AJPH.2014.302430 25790408 PMC4566563

[41] Chilton MM, Rabinowich JR, Woolf NH. Very low food security in the USA is linked with exposure to violence. Public Health Nutr. 2014;17(1):73–82. 10.1017/S1368980013000281 23432921 PMC10282483

[42] Conroy A, Cohen M.H., Frongillo E.A., Tsai A.C., Wilson T.E., Wentz E.L., et al. Food insecurity and violence in a prospective cohort of US women at risk or living with HIV. Under review. 2018. 10.1371/journal.pone.0213365 PMC6402690 30840700

[43] Conroy AA, Cohen MH, Frongillo EA, Tsai AC, Wilson TE, Wentz EL, et al. Food insecurity and violence in a prospective cohort of women at risk for or living with HIV in the U.S. PLoS One. 2019;14(3):e0213365. 10.1371/journal.pone.0213365 30840700 PMC6402690

[44] Hernandez DC, Marshall A, Mineo C. Maternal depression mediates the association between intimate partner violence and food insecurity. J Womens Health (Larchmt). 2014;23(1):29–37. 10.1089/jwh.2012.4224 24131321 PMC3880922

[45] Chilton M, Booth S. Hunger of the body and hunger of the mind: African American women’s perceptions of food insecurity, health and violence. J Nutr Educ Behav. 2007;39(3):116–25. 10.1016/j.jneb.2006.11.005 17493561

[46] Hatcher AM, Stockl H, McBride RS, Khumalo M, Christofides N. Pathways From Food Insecurity to Intimate Partner Violence Perpetration Among Peri-Urban Men in South Africa. Am J Prev Med. 2019;56(5):765–72. 10.1016/j.amepre.2018.12.013 30905482

[47] Buller AM, Hidrobo M, Peterman A, Heise L. The way to a man’s heart is through his stomach?: a mixed methods study on causal mechanisms through which cash and in-kind food transfers decreased intimate partner violence. BMC Public Health. 2016;16:488. 10.1186/s12889-016-3129-3 27278935 PMC4898371

[48] Laurenzi C, Field S, Honikman S. Food Insecurity, Maternal Mental Health, and Domestic Violence: A Call for a Syndemic Approach to Research and Interventions. Matern Child Health J. 2020;24(4):401–4. 10.1007/s10995-019-02872-8 32009230

[49] Singer M. A dose of drugs, a touch of violence, a case of AIDS: conceptualizing the SAVA syndemic. Free Inq Creativ Sociol. 1996;24(2):99–110.

[50] Singer M, Bulled N, Ostrach B, Mendenhall E. Syndemics and the biosocial conception of health. Lancet. 2017;389(10072):941–50. 10.1016/S0140-6736(17)30003-X 28271845

[51] Tsai AC, Burns BF. Syndemics of psychosocial problems and HIV risk: A systematic review of empirical tests of the disease interaction concept. Soc Sci Med. 2015;139:26–35. 10.1016/j.socscimed.2015.06.024 26150065 PMC4519429

[52] Coleman-Jensen A, Rabbitt M.P., Gregory C.A., Singh A. Household food security in the United States in 2019. 2020.

[53] Feeding America. Understand food insecurity 2021 [Available from: https://hungerandhealth.feedingamerica.org/understand-food-insecurity/.

[54] Weiser SD, Young SL, Cohen CR, Kushel MB, Tsai AC, Tien PC, et al. Conceptual framework for understanding the bidirectional links between food insecurity and HIV/AIDS. Am J Clin Nutr. 2011;94(6):1729S–39S. 10.3945/ajcn.111.012070 22089434 PMC3226026

[55] Power EM. Economic abuse and intra-household inequities in food security. Can J Public Health. 2006;97(3):258–60. 10.1007/BF03405600 16827421 PMC6975889

[56] Gundersen C, Ziliak JP. Food Insecurity And Health Outcomes. Health Aff (Millwood). 2015;34(11):1830–9. 10.1377/hlthaff.2015.0645 26526240

[57] Adimora AA, Ramirez C, Benning L, Greenblatt RM, Kempf MC, Tien PC, et al. Cohort Profile: The Women’s Interagency HIV Study (WIHS). Int J Epidemiol. 2018;47(2):393–4i. 10.1093/ije/dyy021 29688497 PMC5913596

[58] Urban Institute. Public Welfare Expenditures 2020 [Available from: https://www.urban.org/policy-centers/cross-center-initiatives/state-and-local-finance-initiative/state-and-local-backgrounders/public-welfare-expenditures.

[59] Frongillo EA Jr. Validation of measures of food insecurity and hunger. J Nutr. 1999;129(2S Suppl):506S–9S. 10.1093/jn/129.2.506S 10064319

[60] Whittle HJ, Palar K, Ranadive NA, Turan JM, Kushel M, Weiser SD. "The land of the sick and the land of the healthy": Disability, bureaucracy, and stigma among people living with poverty and chronic illness in the United States. Soc Sci Med. 2017;190:181–9. 10.1016/j.socscimed.2017.08.031 28865254 PMC5937915

[61] Whittle HJ, Palar K, Seligman HK, Napoles T, Frongillo EA, Weiser SD. How food insecurity contributes to poor HIV health outcomes: Qualitative evidence from the San Francisco Bay Area. Soc Sci Med. 2016;170:228–36. 10.1016/j.socscimed.2016.09.040 27771206

[62] Krippendorff K. Content analysis: an introduction to its methodology. 3rd ed. Los Angeles; London: SAGE; 2013. xiv, 441 p. p.

[63] Bradley EH, Curry LA, Devers KJ. Qualitative data analysis for health services research: developing taxonomy, themes, and theory. Health Serv Res. 2007;42(4):1758–72. 10.1111/j.1475-6773.2006.00684.x 17286625 PMC1955280

[64] United States Department of Agriculture (USDA). How are food security and insecurity measured? 2020 [Available from: https://www.ers.usda.gov/topics/food-nutrition-assistance/food-security-in-the-us/measurement/#measurement.

[65] Bailey ZD, Krieger N, Agenor M, Graves J, Linos N, Bassett MT. Structural racism and health inequities in the USA: evidence and interventions. Lancet. 2017;389(10077):1453–63. 10.1016/S0140-6736(17)30569-X 28402827

[66] Feagin J, Bennefield Z. Systemic racism and U.S. health care. Soc Sci Med. 2014;103:7–14. 10.1016/j.socscimed.2013.09.006 24507906

[67] Czeisler ME, Lane RI, Petrosky E, Wiley JF, Christensen A, Njai R, et al. Mental Health, Substance Use, and Suicidal Ideation During the COVID-19 Pandemic—United States, June 24–30, 2020. MMWR Morb Mortal Wkly Rep. 2020;69(32):1049–57. 10.15585/mmwr.mm6932a1 32790653 PMC7440121

[68] Schanzenbach DW, Pitts, A. How much has food insecurity risen? Evidence from the Census Household Pulse Survey: Institute for Policy Research Rapid Research Report; 2020 [Available from: https://www.ipr.northwestern.edu/documents/reports/ipr-rapid-researchreports-pulse-hh-data-10-june-2020.pdf.

[69] Gosangi B, Park H, Thomas R, Gujrathi R, Bay CP, Raja AS, et al. Exacerbation of Physical Intimate Partner Violence during COVID-19 Lockdown. Radiology. 2020:202866. 10.1148/radiol.2020202866 PMC7427119 32787700

[70] Spinelli MA, Hickey MD, Glidden DV, Nguyen JQ, Oskarsson JJ, Havlir D, et al. Viral suppression rates in a safety-net HIV clinic in San Francisco destabilized during COVID-19. AIDS. 2020;34(15):2328–31. 10.1097/QAD.0000000000002677 32910069 PMC7674255

[71] Whittle HJ, Sheira LA, Wolfe WR, Frongillo EA, Palar K, Merenstein D, et al. Food insecurity is associated with anxiety, stress, and symptoms of posttraumatic stress disorder in a cohort of women with or at risk of HIV in the United States. J Nutr. 2019;149(8):1393–403. 10.1093/jn/nxz093 31127819 PMC6675617

[72] Rees S, Silove D, Chey T, Ivancic L, Steel Z, Creamer M, et al. Lifetime prevalence of gender-based violence in women and the relationship with mental disorders and psychosocial function. JAMA. 2011;306(5):513–21. 10.1001/jama.2011.1098 21813429

[73] Nagata JM, Palar K, Gooding HC, Garber AK, Whittle HJ, Bibbins-Domingo K, et al. Food Insecurity Is Associated With Poorer Mental Health and Sleep Outcomes in Young Adults. J Adolesc Health. 2019;65(6):805–11. 10.1016/j.jadohealth.2019.08.010 31587956 PMC6874757

[74] Schwartz RM, Weber KM, Schechter GE, Connors NC, Gousse Y, Young MA, et al. Psychosocial correlates of gender-based violence among HIV-infected and HIV-uninfected women in three US cities. AIDS Patient Care STDS. 2014;28(5):260–7. 10.1089/apc.2013.0342 24724987 PMC4011431

[75] Whittle HJ, Sheira LA, Frongillo EA, Palar K, Cohen J, Merenstein D, et al. Longitudinal associations between food insecurity and substance use in a cohort of women with or at risk for HIV in the United States. Addiction. 2019;114(1):127–36. 10.1111/add.14418 30109752 PMC6516859

[76] Jacobsen LK, Southwick SM, Kosten TR. Substance use disorders in patients with posttraumatic stress disorder: a review of the literature. Am J Psychiatry. 2001;158(8):1184–90. 10.1176/appi.ajp.158.8.1184 11481147

[77] Conner KR, Pinquart M, Gamble SA. Meta-analysis of depression and substance use among individuals with alcohol use disorders. J Subst Abuse Treat. 2009;37(2):127–37. 10.1016/j.jsat.2008.11.007 19150207 PMC4864601

[78] Hutton HE, Lyketsos CG, Zenilman JM, Thompson RE, Erbelding EJ. Depression and HIV risk behaviors among patients in a sexually transmitted disease clinic. Am J Psychiatry. 2004;161(5):912–4. 10.1176/appi.ajp.161.5.912 15121659

[79] El-Bassel N, Gilbert L, Vinocur D, Chang M, Wu E. Posttraumatic stress disorder and HIV risk among poor, inner-city women receiving care in an emergency department. Am J Public Health. 2011;101(1):120–7. 10.2105/AJPH.2009.181842 21088271 PMC3000708

[80] Booth RE, Watters JK, Chitwood DD. HIV risk-related sex behaviors among injection drug users, crack smokers, and injection drug users who smoke crack. Am J Public Health. 1993;83(8):1144–8. 10.2105/ajph.83.8.1144 8342724 PMC1695160

[81] Gonzalez JS, Batchelder AW, Psaros C, Safren SA. Depression and HIV/AIDS treatment nonadherence: a review and meta-analysis. J Acquir Immune Defic Syndr. 2011;58(2):181–7. 10.1097/QAI.0b013e31822d490a 21857529 PMC3858003

[82] Vranceanu AM, Safren SA, Lu M, Coady WM, Skolnik PR, Rogers WH, et al. The relationship of post-traumatic stress disorder and depression to antiretroviral medication adherence in persons with HIV. AIDS Patient Care STDS. 2008;22(4):313–21. 10.1089/apc.2007.0069 18338960

[83] Gonzalez A, Mimiaga MJ, Israel J, Andres Bedoya C, Safren SA. Substance use predictors of poor medication adherence: the role of substance use coping among HIV-infected patients in opioid dependence treatment. AIDS Behav. 2013;17(1):168–73. 10.1007/s10461-012-0319-6 23008124 PMC3632258

[84] Meyer JP, Springer SA, Altice FL. Substance abuse, violence, and HIV in women: a literature review of the syndemic. J Womens Health (Larchmt). 2011;20(7):991–1006. 21668380 10.1089/jwh.2010.2328 PMC3130513

[85] Illangasekare S, Burke J, Chander G, Gielen A. The syndemic effects of intimate partner violence, HIV/AIDS, and substance abuse on depression among low-income urban women. J Urban Health. 2013;90(5):934–47. 10.1007/s11524-013-9797-8 23529665 PMC3795184

[86] Minkoff-Zern L. Hunger amidst plenty: Farmworker food insecurity and coping strategies in California. Local Environment. 2012;19(2):204–19.

[87] Zadnik E, Sabina C, Cuevas CA. Violence Against Latinas: The Effects of Undocumented Status on Rates of Victimization and Help-Seeking. J Interpers Violence. 2016;31(6):1141–53. 10.1177/0886260514564062 25540190

[88] Peitzmeier SM, Malik M, Kattari SK, Marrow E, Stephenson R, Agenor M, et al. Intimate Partner Violence in Transgender Populations: Systematic Review and Meta-analysis of Prevalence and Correlates. Am J Public Health. 2020;110(9):e1–e14. 10.2105/AJPH.2020.305774 32673114 PMC7427218

[89] Baral SD, Poteat T, Stromdahl S, Wirtz AL, Guadamuz TE, Beyrer C. Worldwide burden of HIV in transgender women: a systematic review and meta-analysis. Lancet Infect Dis. 2013;13(3):214–22. 10.1016/S1473-3099(12)70315-8 23260128

[90] Bockting WO, Miner MH, Swinburne Romine RE, Hamilton A, Coleman E. Stigma, mental health, and resilience in an online sample of the US transgender population. Am J Public Health. 2013;103(5):943–51. 10.2105/AJPH.2013.301241 23488522 PMC3698807

[91] Brennan J, Kuhns LM, Johnson AK, Belzer M, Wilson EC, Garofalo R, et al. Syndemic theory and HIV-related risk among young transgender women: the role of multiple, co-occurring health problems and social marginalization. Am J Public Health. 2012;102(9):1751–7. 10.2105/AJPH.2011.300433 22873480 PMC3416048

[92] Russomanno J, Jabson Tree JM. Food insecurity and food pantry use among transgender and gender non-conforming people in the Southeast United States. BMC Public Health. 2020;20(1):590. 10.1186/s12889-020-08684-8 32349699 PMC7191729

[93] Sandelowski M. The problem of rigor in qualitative research. ANSAdvances in nursing science. 1986;8(3):27–37. 10.1097/00012272-198604000-00005 3083765

[94] U.S. Department of Agriculture. Biden-Harris administration’s actions to reduce food insecurity amid the COVID-19 crisis 2021 [Available from: https://www.usda.gov/media/press-releases/2021/03/03/biden-harris-administrations-actions-reduce-food-insecurity-amid.

[95] Collins LF, Sheth AN, Mehta CC, Naggie S, Golub ET, Anastos K, et al. The Prevalence and Burden of Non-AIDS Comorbidities among Women living with or at-risk for HIV Infection in the United States. Clin Infect Dis. 2020. 10.1093/cid/ciaa204 PMC8075036 32115628

[96] Keith-Jennings B, Llobrera J, Dean S. Links of the Supplemental Nutrition Assistance Program With Food Insecurity, Poverty, and Health: Evidence and Potential. Am J Public Health. 2019;109(12):1636–40. 10.2105/AJPH.2019.305325 31693420 PMC6836787

[97] SAMHSA’s Trauma and Justice Strategic Initiative. SAMHSA’s Concept of Trauma and Guidance for a Trauma-Informed Approach 2014 [Available from: https://ncsacw.samhsa.gov/userfiles/files/SAMHSA_Trauma.pdf.

[98] Morrissey JP, Jackson EW, Ellis AR, Amaro H, Brown VB, Najavits LM. Twelve-month outcomes of trauma-informed interventions for women with co-occurring disorders. Psychiatr Serv. 2005;56(10):1213–22. 10.1176/appi.ps.56.10.1213 16215186

[99] Cocozza JJ, Jackson EW, Hennigan K, Morrissey JP, Reed BG, Fallot R, et al. Outcomes for women with co-occurring disorders and trauma: program-level effects. J Subst Abuse Treat. 2005;28(2):109–19. 10.1016/j.jsat.2004.08.010 15780540

[100] Machtinger EL, Cuca YP, Khanna N, Rose CD, Kimberg LS. From treatment to healing: the promise of trauma-informed primary care. Womens Health Issues. 2015;25(3):193–7. 10.1016/j.whi.2015.03.008 25965151

